# Similarity analysis between species of the genus *Quercus* L. (Fagaceae) in southern Italy based on the fractal dimension

**DOI:** 10.3897/phytokeys.113.30330

**Published:** 2018-12-11

**Authors:** Musarella Carmelo Maria, Cano-Ortiz Ana, Piñar Fuentes José C rlos, Navas-Ureña uan, Pinto Gomes Carlos José, Quinto-Canas Ricardo, Cano Eusebio, Spampinato Giovanni

**Affiliations:** 1 Dpt. of Animal and Plant Biology and Ecology, Section of Botany, University of Jaén, Campus Universitario Las Lagunillas s/n. 23071, Jaén, Spain; 2 Dpt. of AGRARIA, “Mediterranea” University of Reggio Calabria, Località Feo di Vito, 89122 Reggio Calabria, Italy; 3 Dpt. of Mathematics, Applied Mathematics area, University of Jaén, Campus Universitario Las Lagunillas s/n. 23071, Jaén, Spain; 4 Dpt. of Landscape, Environment and Planning/Institute of Mediterranean Agricultural and Environmental Sciences (ICAAM), University of Évora, Rua Romão Ramalho, Portugal; 5 Faculty of Sciences and Technology, University of Algarve, Campus de Gambelas, 8005-139 Faro, Portugal; 6 Centre of Marine Sciences (CCMAR), University of Algarve, Campus de Gambelas, 8005-139 Faro, Portugal

**Keywords:** deciduous, dimension, fractal analysis, phenotype, sclerophyllous, species, Calabria

## Abstract

The fractal dimension (FD) is calculated for seven species of the genus *Quercus* L. in Calabria region (southern Italy), five of which have a marcescent-deciduous and two a sclerophyllous character. The fractal analysis applied to the leaves reveals different FD values for the two groups. The difference between the means and medians is very small in the case of the marcescent-deciduous group and very large when these differences are established between both groups: all this highlights the distance between the two groups in terms of similarity. Specifically, *Q.crenata*, which is hybridogenic in origin and whose parental species are *Q.cerris* and *Q.suber*, is more closely related to *Q.cerris* than to *Q.suber*, as also expressed in the molecular analysis. We consider that, in combination with other morphological, physiological and genetic parameters, the fractal dimension is a useful tool for studying similarities amongst species.

## Introduction

*Quercus* L. is an important genus containing several species of trees dominating different forest communities. The ecological and economic role of *Quercus* spp. is well known (Quinto-Canas et al. 2010, 2018, Vila-Viçosa et al. 2015, Piñar Fuentes et al. 2017, Spampinato et al. 2016, 2017, Vessella et al. 2017). Some species (such as cork oak) are specifically very useful for carbon sequestration and as raw materials for a post carbon city (Del Giudice et al. 2019, De Paola et al. 2019, Malerba et al. 2019, Massimo et al. 2019, Spampinato et al. 2019).

In the genus *Quercus* have been counted between 300 (Lawrence 1951, Elias 1971) and 600 species (Soepadmo 1972). However, several inventories (Schwarz 1964, Nixon 1993, Valencia 2004, Menitsky 2005) amount between 396 and 430 species for this critical genus. According to Musarella and Spampinato (2012a,b) in Calabria region (Southern Italy), there are 11 taxa: QuercusilexL.subsp.ilex, *Q.suber* L., *Q.congesta* C.Presl., *Q.cerris* L., *Q.frainetto* Ten., Q.roburL.subsp.brutia (Ten.) O.Schwarz., *Q.virgiliana* (Ten.) Ten., *Q.amplifolia* Guss., *Q.dalechampii* Ten., *Q.crenata* Lam. and Q.petraea(Matt.)Liebl.subsp.austrotyrrhenica Brullo, Guarino & Siracusa. Bartolucci et al. (2018) record 17 taxa for Italy (9 of these sure for Calabria). Unfortunately, these authors do not consider in their checklist some species, such as *Q.virgiliana* and *Q.crenata*. However, we consider that *Q.virgiliana* is present in Calabria and it is clearly distinct from Q.pubescensWilld.subsp.pubescens according to Brullo et al. (1999), Viscosi et al. (2011) and Brullo and Guarino (2017). This species plays a very important role in the forest vegetation of the region (Brullo et al. 2001) and characterises the habitat 91AA*: Eastern white oak woods (AA.VV 2013, Biondi et al. 2009) distributed in Italy and in the Balkan Peninsula. Moreover, we consider *Q.crenata* as a species of hybrid origin from *Q.cerris* and *Q.suber*, according to Conte et al. (2007) and Brullo and Guarino (2017).

Leaf morphology has been studied throughout the history of botany, using leaf shape, edge, vein arrangement, hairiness and other features as important characters in systematics (Coutinho 1939, Amaral Franco 1990). Species have been described by means of the analysis of the size and shape of several leaf characters and using biometric studies. Morphometry and the leaf vascular system have traditionally been key aspects for establishing the description and biometrics of the species; in morphometry, the leaf shape and edge and the arrangement of the veins are all common systematic characters used to characterise different species. For a correct determination of each species and their hybrids, their taxonomic characters must be observed with specific instruments, e.g. powerful microscopes capable of highlighting micromorphometric characters (Vila-Viçosa et al. 2014).

Numerous authors have noted the comparative inaccuracy of early descriptive and biometric studies (Mouton 1970, 1976, Hickey and Wolfe 1975, Hickey 1979). Classic descriptive methods do not establish clear differences between pure individuals and their hybrids, so molecular studies are proposed for pure and hybrid strains (Conte et al. 2007, Curtu et al. 2007, Coutinho et al. 2014, 2015). More precise biometric studies subsequently emerged that allowed a more meticulous representation of the leaf detail or the other parts of the plants (e.g. Cano et al. 2017). Biometrics thus came into its own for pinpointing the differences between species and taxonomic groups.

In their study of several *Quercus* species, Camarero et al. (2003) and Fortini et al. (2015) analysed the leaf morphology for pure and hybridogenic populations and observed the variability of their morphological characters. These phenotypical characters must be precisely quantified to establish the differences between pure species and their hybrids, which can be recognised through fractal analysis.

We calculated the fractal dimension by the box-counting method integrated in the ImageJ software (Abramoff et al. 2004), as it allows the possibility of assessing the fractal dimension of structures that are not totally self-similar. To resolve the controversy regarding certain species/subspecies in the genus *Quercus*, a discriminant analysis is required that can clearly differentiate the species/subspecies and the degree of relationship between them. The fractal dimension, which has not so far been widely applied in botany, although somewhat more so in medicine, was used for this purpose (Esteban et al. 2007, 2009, Lopes and Beltrouni 2009).

The main aim of this work is to establish an analysis of similarity of leaf shape amongst seven species in the genus *Quercus* from Italy and corroborate our previous studies (Musarella et al. 2013), in which we proposed a FD < 1.6 for sclerophyllous *Quercus* and FD > 1.6 for deciduous and marcescent *Quercus*.

## Methods

### Data collection

In this work, we analysed 7 species living in Calabria using 275 tree samples belonging to Quercusrobursubsp.brutia, *Q.cerris*, *Q.congesta*, *Q.crenata*, Q.ilexsubsp.ilex, *Q.suber* and *Q.virgiliana*. Orientation largely determines the amount of light the leaves receive for photosynthesis and their size can thus be affected by this greater or lesser exposure to light. For this reason, samples were taken from the four cardinal points on each tree to examine the possible influence of orientation on leaf development. A total of 1,099 leaves were analysed from 120 samples of Q.robursubsp.brutia, 120 from *Q.cerris*, 154 from *Q.congesta*, 147 from *Q.crenata*, 240 from Q.ilexsubsp.ilex, 139 from *Q.suber* and 179 from *Q.virgiliana*. All the leaves were colour-scanned in a scanner with a resolution of 1200 dpi and 24-bit colour. After scanning, the leaf was transformed to image 8-bit greyscales and the image was segmented by selecting the greyscale between 111 and 126. We opened this image with the ImageJ programme in order to determine its fractal dimension (FD).

### The fractal dimension (FD)

Fractal geometry is the most suitable method for characterising the complexity of the vascular system or other mathematically similar structures such as stream drainage networks in chicken embryos or the distribution of the vascular system of a leaf (Horton 1945, Vigo et al. 1998). De Araujo Mariath et al. (2010) developed a method using digital images of leaves to determine the fractal dimensions of the leaf vascular system in three species of *Relbunium* (Endl.) Hook. F. (Rubiaceae), with the aim of quantifying and determining its complexity so it could be used as a taxonomic character. Recently, Cuzzocrea et al. (2017) described an algorithm to estimate the parameters of Iterated Function System (IFS) fractal models, using IFS to model speech and electroencephalographic signals and to compare the results.

All man-made objects can be described in simple shapes using Euclidean geometry. However, natural objects have irregular forms that cannot always be represented using this method (Glenny et al. 1985).

Due to the recentness of the discovery and its wide range of applications, there is still no universal definition of what actually constitutes a fractal. They are thus described according to their common properties: specifically, they must have the same appearance at any scale of observation, meaning that a fractal object can be broken down into parts, each of which is identical to the whole object (self-affinity or self-similarity); they must have a fractional and not a whole dimension (fractal dimension); and finally the relationship between two of their variables must be a power law (where the exponent is its fractal dimension, Mandelbrot 1983). Topological and Euclidean dimensions cannot be applied to highly irregular objects such as coastlines. Mandelbrot (1967) published a widely-referenced work where he proved that it was impossible to give an exact value of the length of the coast, as this measurement depended on the unit of scale used. Thus in the case of irregular curves, a small FD of close to 1 signifies a low level of complexity, whereas values close to 2 indicate a very high level of irregularity.

**Figure 1.**
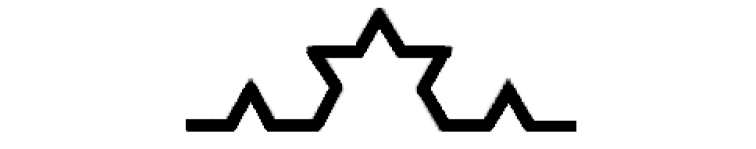
The Koch curve.

When an object is totally self-similar, such as the mathematical fractal known by the name of the Koch curve (Figure 1), the dimension used is known as the self-similarity dimension.

A unit segment can be divided – for example – into three pieces similar to the original, each with a length of 1/3. In general, where *N(h)* is the number of pieces with a length *h*, it follows that *N(h)* ∙ *h*^1^ = 1. If we now look at a square with a unit side, we can break it down into 9 = 3^2^ smaller squares with a side of ⅓; that is to say *N(h)* ∙ *h*^2^ = 1. Finally, in the case of a cube, it is easy to see that the following is true: *N(h)* ∙ *h*^3^ = 1. That is, the exponent of *h* coincides with the topological and Euclidean dimension of the straight line (1), the square (2) and the cube (3) (Martinez Bruno and de Oliveira Plotze 2008).

By extrapolation from this concept, if the object is completely self-similar, there is a relationship between the scale factor *h* and the number of pieces *N(h)* into which the object can be divided, which is given by *N(h)* = (1/*h*)*^D^*; that is to say


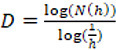
.

Thus the fractal dimension of the Koch curve is:


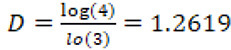
,

a number that is very similar to the FD of the English coastline.

However, natural objects like leaves are not perfect fractals, as they are not totally self-similar but are said to be statistically similar. In this case, the value of their fractal dimension is known by the name of Hausdorff-Besicovitch and is:


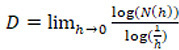
.

The calculation of this limit is somewhat complicated and requires the use of different algorithms such as dilation methods, the perimeter method, Grassberger and Procaccia’s correlation dimension and box-counting method. This last is the most widely used as it is very simple to implement with computer technology and highly accurate (Glenny et al. 1985, Jian Li et al. 2009).

To find the fractal dimension of a digital image using the box-counting method (Mandelbrot 1983), the image must be transformed into black (the leaf) and white (the background). A grid is then superimposed on the image and the number of times the leaf intersects a grid square is counted. The image is covered with a grid of squares initially with side 2 and subsequently with squares with side 3, 4, 6, 8, 12, 16 and 32 (in Table 1; C2, C3, C4, C6, C8, C12, C16 and C32). The side of square *h* is then reduced and the logarithm of the number of intersections *N(h)* is represented based on the logarithm of the inverse function of the side. The dimension of the object coincides with the slope of the regression line defined by the point cluster *(log(1/h), log(N(h))* produced when the value of the side of the grid square is changed.

**Table 1.** Number of boxes occupied for each box size.

**Label**	**C2**	**C3**	**C4**	**C6**	**C8**	**C12**	**C16**	**C32**	**D**
QCONGESTA1_E_01	358874	166858	97125	44308	25268	11452	6553	1727	1.93

The graphic representation of the regression line and the point cluster shows two very clearly differentiated parts. The minimum and maximum box size is therefore very important when applying this method. In fact, the approximation error must be reduced by selecting points with a “more linear” form as a box size.

### Calculating the fractal dimension (FD)

**Figure 2.**
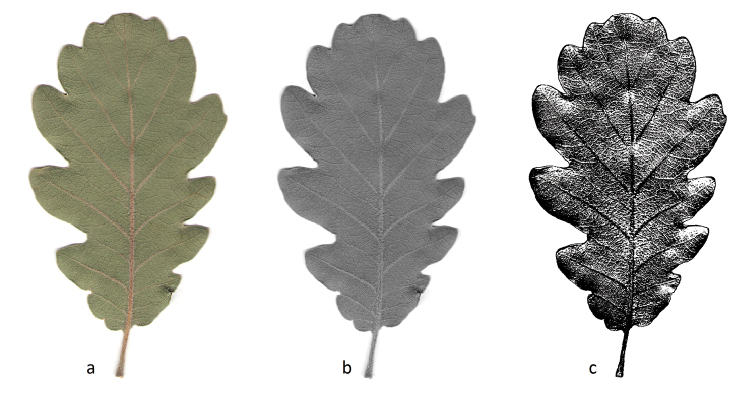
**a** RGB colour image **b** 8-bit greyscale image and **c** binary selection of an image of a *Quercuscrenata* leaf.

The FD was calculated by the box-counting method (Esteban et al. 2007) using the free software ImageJ version 1.47 (http://imagej.com). The digital image of the leaf in RGB colour (Figure 2a) was first converted into an 8-bit image (Figure 2b) where each pixel was represented with a greyscale from 1 to 256. In order to select the most important information, the image was subsequently segmented to produce a greyscale between 111 and 126 and then converted into binary so the leaf takes the value 1 and the rest the value 0 (Figure 2c).

The box-counting algorithm was then applied to this black-and-white image of the venation network of the leaf to calculate the FD with box sizes (*h*) ranging from 2 to 32. Specifically, the image is covered with a grid of squares initially with side 2 and subsequently with squares with sides 3, 4, 6, 8, 12, 16 and 32 (in the image C2, C3, C4, C6, C8, C12, C16 and C32). Table 1 shows the number of boxes occupied *(N(h)*) for each box size.

**Figure 3.**
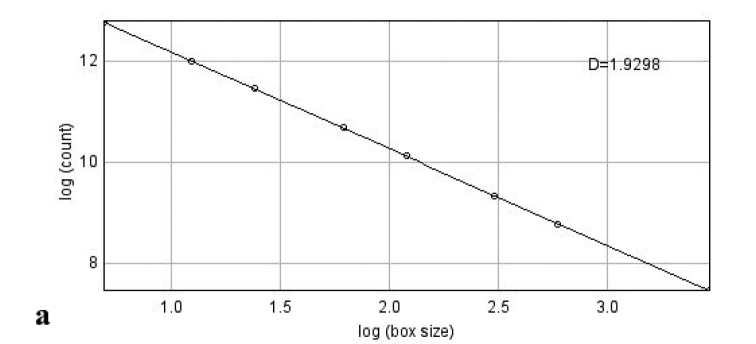
Regression line for the points (log(1/h), log(N(h)).

Once the points were represented *(log(1/h)*, *log(N(h))*, we calculated the regression line (Figure 3) whose slope corresponds to the value of the fractal dimension; in our case, the FD=1.9298, Standard Error= 0.0044, p-Value=1.01384*10^(-14). As can be seen in the graph, the fit is fairly good as the points are very close to the resulting regression line.

For the statistical treatment, the mean FDs were obtained for each species and an analysis of variance was undertaken to test for significant differences amongst the means. First, the Shapiro-Wilk normality test and the difference between the mean, median and kurtosis indicate that our data do not follow a normal distribution (Table 2), meaning non-parametric methods must be used. To determine whether orientation affects the leaf morphological character, we applied a non-parametric Kruskal-Wallis test which, based on the medians, compares the leaves from the same population and from the four orientations. We also applied the standardised kurtosis coefficient to determine whether there is significant normality in the data. In the case of significant differences in the analysis of variance, we applied the LSD (Least Significant Difference) multiple comparison test.

**Table 2.** Descriptive statistics of FD values for each species and orientation.

	Taxa	Median	Mean	Variance (n-1)	Kurtosis (Pearson)	St. root of the variance	St. root [kurtosis (Fisher)]
North	Q. robur subsp. brutia	1.5440	1.5290	0.0730	-1.3300	0.0192	0.8327
	* Q. cerris *	1.6760	1.6676	0.0375	-0.5768	0.0098	0.8327
	* Q. congesta *	1.8780	1.8310	0.0138	1.8836	0.0032	0.7587
	* Q. crenata *	1.9195	1.8669	0.0172	6.2735	0.0040	0.7497
	Q. ilex subsp. ilex	1.3530	1.3804	0.0297	0.9245	0.0055	0.6133
	* Q. suber *	0.8620	0.9001	0.0703	0.3360	0.0173	0.7879
	* Q. virgiliana *	1.9310	1.9192	0.0016	7.4011	0.0003	0.6876
South	Q. robur subsp. brutia	1.7675	1.6220	0.0895	-1.6597	0.0235	0.8327
	* Q. cerris *	1.6600	1.6190	0.0337	0.3597	0.0089	0.8327
	* Q. congesta *	1.9000	1.8749	0.0058	2.8406	0.0014	0.7587
	* Q. crenata *	1.9200	1.8803	0.0106	2.8957	0.0025	0.7497
	Q. ilex subsp. ilex	1.3610	1.3442	0.0149	-0.2129	0.0028	0.6133
	* Q. suber *	0.9395	0.9487	0.0408	0.0321	0.0100	0.7879
	* Q. virgiliana *	1.9120	1.8780	0.0060	1.1207	0.0013	0.6876
East	Q. robur subsp. brutia	1.8405	1.7336	0.0428	-0.2321	0.0112	0.8327
	* Q. cerris *	1.8360	1.8110	0.0143	-0.0039	0.0037	0.8327
	* Q. congesta *	1.9230	1.9215	0.0008	2.2392	0.0002	0.7587
	* Q. crenata *	1.9270	1.8476	0.0257	1.2883	0.0060	0.7497
	Q. ilex subsp. ilex	1.3170	1.2954	0.0196	1.7224	0.0036	0.6133
	* Q. suber *	0.8850	0.9059	0.0475	-0.3256	0.0117	0.7879
	* Q. virgiliana *	1.9445	1.9287	0.0032	11.3639	0.0007	0.6876
West	Q. robur subsp. brutia	1.5715	1.5676	0.0800	-1.2799	0.0210	0.8327
	* Q. cerris *	1.6050	1.6116	0.0643	2.0300	0.0169	0.8327
	* Q. congesta *	1.9180	1.8985	0.0030	0.4157	0.0007	0.7587
	* Q. crenata *	1.9030	1.8754	0.0085	2.8668	0.0020	0.7497
	Q. ilex subsp. ilex	1.4170	1.4302	0.0429	0.1534	0.0080	0.6133
	* Q. suber *	0.9535	0.9746	0.0615	0.2308	0.0151	0.7879
	* Q. virgiliana *	1.9440	1.9317	0.0015	6.6553	0.0003	0.6876
Mean	Q. robur subsp. brutia	1.5500	1.6130	0.0493	-1.6202	0.0129	138.47.00
	* Q. cerris *	1.7029	1.6773	0.0253	1.8675	0.0066	0.8327
	* Q. congesta *	1.8960	1.8815	0.0026	-0.6569	0.0006	0.7587
	* Q. crenata *	1.8866	1.8675	0.0052	0.9650	0.0012	0.7497
	Q. ilex subsp. ilex	1.3625	1.3625	0.0053	-0.4868	0.0010	0.6133
	* Q. suber *	0.9164	0.9323	0.0267	-0.1453	0.0066	0.7879
	* Q. virgiliana *	1.9184	1.9144	0.0007	-0.9555	0.0001	0.6876

In the hypothetical case that the difference between the fractal values (means and medians) for two species is zero or has a quotient of one, the degree of relationship between the two species is 100%; DfA – DfB = 0; DfA / DfB = 1, species A and B are equal; thus the lower the fractal difference or the nearer the fractal quotient is to 1, the greater the similarity between the species.

## Results

The analysis of the FD values for each orientation and for each species shows that for Q.robursubsp.brutia, *Q.cerris*, *Q.congesta* and *Q.virgiliana*, the orientation influences the values of FD, as there are significant differences for these species (Table 3).

**Table 3.** Kruskal-Wallis analysis for the values of FD in each orientation for each of the species. In bold: the significant values for which orientation influences the FD at 95% confidence.

Kruskal-Wallis:	Q. robur subsp. brutia	* Q. cerris *	* Q. congesta *	* Q. crenata *	Q. ilex subsp. ilex	* Q. suber *	* Q. virgiliana *
Mean North	1.5290	1.6676	1.8310	1.8669	1.3804	0.9001	1.9192
Mean South	1.6220	1.6190	1.8749	1.8803	1.3442	0.9487	1.8780
Mean East	1.7336	1.8110	1.9215	1.8476	1.3276	0.9059	1.9287
Mean West	1.5676	1.6116	1.8985	1.8754	1.3895	0.9746	1.9317
St. Deviation North	0.2702	0.1936	0.1174	0.1313	0.1723	0.2651	0.0406
St. Deviation South	0.2992	0.1836	0.0763	0.1030	0.1220	0.2020	0.0777
St. Deviation East	0.2069	0.1194	0.0288	0.1602	0.1074	0.2180	0.0563
St. Deviation West	0.2829	0.2535	0.0546	0.0921	0.1564	0.2479	0.0392
K (Observed value)	9.9875	20.5115	23.0332	1.6844	8.0795	3.0683	38.4400
K (Critical value)	9.4877	7.8147	7.8147	7.8147	9.4877	7.8147	7.8147
p-value	**0.0406**	**0.0001**	**< 0.0001**	0.6404	0.0887	0.3812	**< 0.0001**

These species correspond to deciduous or marcescent species, whereas the perennial species Q.ilexsubsp.ilex, *Q.suber* and *Q.crenata* do not show significant differences in the values of FD for the different levels of orientation.

An analysis of the average FD values for each species indicates that there are significant differences between the different levels of species under study (Table 4). Subsequently, the Conover-Iman test of multiple comparisons between all pairs shows the pairs of species between which there are significant differences (Table 5).

**Table 4.** Kruskal-Wallis test.

K (Observed value)	220.2702
K (Critical value)	12.5916
GDL	6
p-value (bilateral)	< 0.0001
alpha	0.05

**Table 5.** Differences in FD by pairs between each species (in parentheses, p-value). In bold: significant differences at 95% confidence.

	Q. robur subsp. brutia	* Q. cerris *	* Q. congesta *	* Q. crenata *	Q. ilex subsp. ilex	* Q. suber *	* Q. virgiliana *
Q. robur subsp. brutia	–						
* Q. cerris *	4.26 (0.6392)	–					
* Q. congesta *	**71.21 (<0.0001)**	**66.95 (<0.0001)**	–				
* Q. crenata *	**68.55 (<0.0001)**	**64.29 (<0.0001)**	-2.65 (0.7439)	–			
Q. ilex subsp. ilex	-**58.63 (<0.0001)**	-**62.9 (<0.0001)**	-**129.85 (<0.0001)**	-**127.19 (<0.0001)**	–		
* Q. suber *	-**109.43 (<0.0001)**	-**113.7 (<0.0001)**	-**180.65 (<0.0001)**	-**177.99 (<0.0001)**	-**50.8 (<0.0001)**	–	
* Q. virgiliana *	**96.87 (<0.0001)**	**92.61 (<0.0001)**	**25.66 (0.001)**	**28.32 (0.0002)**	**155.51 (<0.0001)**	**206.31 (<0.0001)**	–

As can be seen in Table 5, there are pairs of species for which there are significant differences in the values of FD. These differences are not only significant between the species Q.robursubsp.brutia - *Q.cerris* and between *Q.crenata* - *Q.congesta*. The fractal dimension is therefore sufficient alone to characterise and separate the species Q.ilexsubsp.ilex, *Q.suber* and *Q.virgiliana*, while the fractal dimension of the vascular network of the leaves calculated by the methodology described does not distinguish Q.robursubsp.brutia from *Q.cerris* and *Q.congesta* from *Q.crenata* on its own.

**Figure 4.**
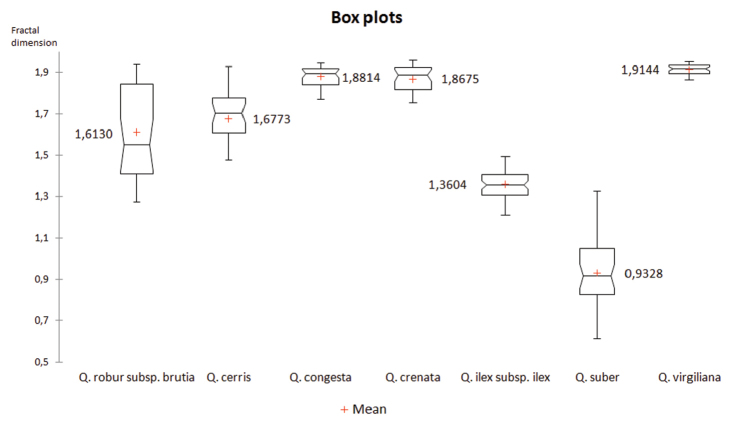
Value of the medians for each homogeneous group. Fractal dimensions (mean values) of the studied species where Quercusilexsubsp.ilex and *Quercussuber* have an FD < 1.6 and the marcescent *Quercus* has a FD > 1.6.

The analysis of the medians of the seven groups (Figure 4) shows that the lowest values of FD correspond to the sclerophyllous *Quercus* species Q.ilexsubsp.ilex and *Q.suber*, whose values are below 1.6, as occurs in the case of the medians. However the marcescent *Quercus* have a median FD of > 1.6; the mean FD values of *Q.suber* and Q.ilexsubsp.ilex are 0.932 and 1.363, respectively, whereas it is 1.613 for the marcescent Q.robursubsp.brutia; 1.677 for *Q.cerris*; 1.881 for *Q.congesta*; 1.868 for *Q.crenata*; and 1.914 for *Q.virgiliana*.

**Figure 5.**
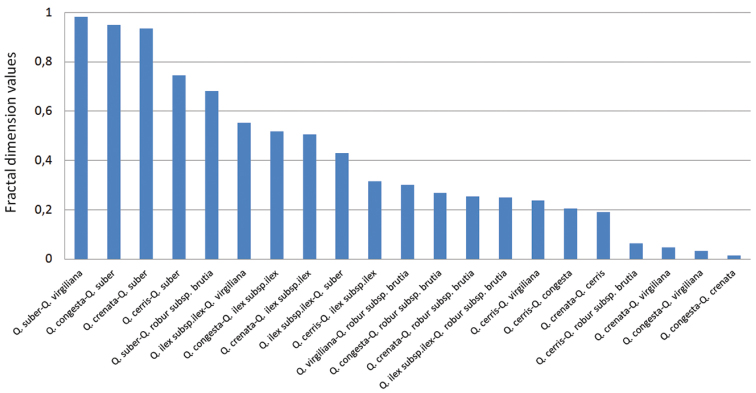
Multiple comparison analysis.

In the multiple comparison analysis (Figure 5) of means and medians, the most significant differences in the two cases are between the sclerophyllous and marcescent *Quercus*, where these differences (means) are 0.982 for *Q.virgiliana-Q.suber* and *0.984 in the case of the medians; however the differences between the marcescent *Quercus* are minimal with *0.015 for *Q.congesta*-*Q.crenata* and *0.188 between *Q.cerris-Q.crenata*. As the value for *Q.crenata-Q.suber* is *0.939, it is evident that *Q.crenata* is more closely related to *Q.cerris* than to *Q.suber* (Figure 5).

In the case of both mean and median values, it is confirmed that the value of the fractal dimension (FD) is less than 1.6 in the case of sclerophyllous *Quercus* and greater for marcescent and deciduous *Quercus* (Figure 4).

The differences between average FD values for marcescent and deciduous *Quercus* species are very low (Table 6). These low differences between average FD values are due to the close similarity between these species. However, there are significant differences in the FD between marcescent and sclerophyllous *Quercus* as they are very distant from each other in evolutionary terms: *Q.virgiliana*-Q.ilexsubsp.ilex 0.551; *Q.virgiliana-Q.suber* 0.982; *Q.congesta*-Q.ilexsubsp.ilex 0.518; *Q.congesta*-*Q.suber* 0.949; *Q.crenata*-Q.ilexsubsp.ilex; 0.505; *Q.crenata*-*Q.suber* 0.936; *Q.cerris*-Q.ilexsubsp.ilex 0.314; *Q.cerris*-*Q.suber* 0.745; and Q.ilexsubsp.ilex-*Q.suber* 0.431.

**Table 6.** Homogeneous character of the groups.

Species	Count	Sum of the ranges	Mean of the ranges	Homogeneous groups				
* Q. suber *	34	626.0000	18.4118	A				
Q. ilex subsp. ilex	59	4083.5000	69.2119		B			
Q. robur subsp. brutia	30	3835.5000	127.8500			C		
* Q. cerris *	30	3963.5000	132.1167			C		
* Q. crenata *	38	7463.5000	196.4079				D	
* Q. congesta *	37	7365.5000	199.0676				D	
* Q. virgiliana *	46	10337.5000	224.7283					E

Based on the differences obtained from FDA–FDB = 0, the most closely related species are: *Q.congesta*-*Q.crenata* 0.023; *Q.cerris*-Q.robursubsp.brutia 0.064; *Q.virgiliana*-*Q.congesta* 0.033; *Q.virgiliana*-*Q.crenata* 0.046; and *Q.crenata*-*Q.cerris* 0.191. The most distant relationship is between *Q.virgiliana*-*Q.suber* 0.982 and *Q.congesta*-*Q.suber* 0.949 (Figure 5).

## Discussion

There is a widespread consensus that complex objects with the same features can be included in the category of fractals. Self-similarity is one of the characteristics of fractal objects, meaning that when these images are broken down into smaller pieces, each one is identical to the whole. The fractional dimension is another of its features.

In the hypothetical case that the difference between the fractal values of two species is zero, or their quotient is one, the degree of relationship between the two species is 100%: Df_A_ – Df_B_ = 0; Df_A_ / Df_B_ = 1, species A and B are equal. Thus the smaller the fractal difference or the closer the fractal quotient is to 1, the greater the similarity between the species; if the value of this quotient is far from 1, as occurs between Df_vi_/Df_su_ > 2, the species *Q.virgiliana* and *Q.suber* are very distant from each other. This occurs when the fractal values are the same and means that the same or similar characters have been measured

Conte et al. (2007) point out the hybridogenic origin of *Q.crenata* and the molecular analysis reveals a closer genetic similarity between *Q.crenata* and *Q.cerris* than between *Q.crenata* and *Q.suber*. The FD of *Q.crenata* is 1.868; for *Q.cerris* it is 1.677; and for *Q.suber* it is 0.932; where Df_Qce_ – Df_Qsu_ = 0.745 and Df_Qce_ / Df_Qsu_ = 1.8, pointing to a large phenotypical (genetic) difference between the parental species. More similarity can be seen between *Q.crenata* and *Q.cerris* than between *Q.crenata* and *Q.suber*, as the difference Df_Qcr_ – Df_Qce_ = 0.191 and Df_Qcr_ / Df_Qce_ = 1.1; they therefore have a high degree of similarity; whereas Df_Qcr_ – Df_Qsu_ = 0.936 and Df_Qcr_ / Df_Qsu_ > 2, indicating substantial phenotypical differences between the hybrid and parental species.

Coutinho et al. (2014, 2015) report a high degree of polymorphism in the genus *Quercus* and establish the molecular analysis of ribosomal DNA through the restriction enzymes to confirm the taxonomic classifications and establish the phylogeny between *Quercus* species. Their results show that the group known as *cerris* contains *Q.crenata* and its parental species *Q.cerris*, whereas it excludes the parental species *Q.suber*; *Q.crenata* is closer to *Q.cerris* with a similarity of 96% compared to a 66% similarity between *Q.suber* and the previous species. Our fractal analysis corroborates the results of Conte et al. (2007) and Coutinho et al. (2015). Curtu et al. (2007) studied four oak species, including *Q.robur* and *Q.cerris* and the intermediate or hybridogenic forms using morphological leaf and genetic markers to classify the hybridisation. In our case, the intermediate or hybrid form corresponds to *Q.crenata* which has its origins in the parental species *Q.cerris* and *Q.suber.* Here the intermediate form *Q.crenata* has a fractal value close to *Q.cerris* and very far from *Q.suber*.

Finally, the orientation has no influence on the fractal dimension between either the same species or between the different species. This means that the shape of the distribution of the leaf vascular network is not affected by possible changes in orientation, thus discounting the effects of environmental variables such as amount of light, temperature, humidity etc., associated with orientation. This evidence is important in *Quercus* species, as in other cases, these environmental variables can influence seed germination and the capacity of some plant species to adapt to extreme environments (Signorino et al. 2011, Musarella et al. 2018, Panuccio et al. 2018, Spampinato et al. 2018): in some cases, the survival or disappearance of a species in an environment may depend on it.

## Conclusions

We confirm that the application of fractal analysis identifies the phenotypical differences between species and can be used as a method to establish their degree of relationship; this is supported by molecular analysis by various authors. In this work we can affirm that sclerophyllous *Quercus* species have a fractal dimension of < 1.6 and marcescent and deciduous *Quercus* species have FD > 1.6; and that *Q.crenata*, a hybrid of *Q.suber* and *Q.cerris*, has a greater similarity to *Q.cerris* than to *Q.suber*. The low values of the mean and median FD revealed by the differences between the FD for marcescent-deciduous *Quercus* species suggest a high degree of similarity amongst the five marcescent-deciduous species. Based on their FD, marcescent *Quercus* species (semideciduous) are more closely related to deciduous than to sclerophyllous *Quercus* species, whereas the sclerophyllous Q.ilexsubsp.ilex and *Q.suber* show substantial morphological differences with the marcescent and deciduous *Quercus* species, as evidenced by fractal analysis. These two species have followed different evolutionary paths from the others, as is to be expected, as the centre of origin of sclerophyllous *Quercus* species is Mediterranean, whereas deciduous *Quercus* species have a temperate origin and marcescent *Quercus* species come from the boundary between the Temperate and Mediterranean bioclimates (Amaral Franco 1990, Sánchez de Dios et al. 2009).
